# Hemodynamic Differences Between Basilar Artery Fenestration and Normal Vertebrobasilar Artery: A Pilot Study

**DOI:** 10.3389/fneur.2021.766174

**Published:** 2022-01-13

**Authors:** Jia Dong, Yuqian Mei, Xuesong Bai, Xinyu Tong, Adam A. Dmytriw, Bin Yang, Tao Wang, Aman B. Patel, Xiaoyan Yang, Mengyue Li, Renjie Yang, Duanduan Chen, Liqun Jiao

**Affiliations:** ^1^Department of Neurosurgery, Xuanwu Hospital, Capital Medical University, Beijing, China; ^2^Interventional Neuroradiology Diagnosis and Treatment Center, Xuanwu Hospital, Capital Medical University, Beijing, China; ^3^School of Life Science, Beijing Institute of Technology, Beijing, China; ^4^School of Medical Imaging, North Sichuan Medical College, Nanchong, China; ^5^China International Neuroscience Institute (China-INI), Beijing, China; ^6^Neuroendovascular Program, Harvard Medical School, Massachusetts General Hospital, Boston, MA, United States; ^7^Department of Radiology, Xuanwu Hospital, Capital Medical University, Beijing, China

**Keywords:** basilar artery fenestration, ischemic stroke, hemodynamic, wall shear stress, computational fluid dynamics

## Abstract

**Background:** Basilar artery fenestration has been proposed as a contributor to ischemic stroke, as unique flow patterns induced by fenestration may be related to thrombus formation or insufficiency. This study aimed to evaluate the hemodynamics of basilar artery fenestration (BAF) using computational fluid dynamics (CFD).

**Methods:** Patients with BAF and normal vertebrobasilar system were recruited and separately evaluated using CFD. Specific geometric vascular models were reconstructed based on 3D-rotational angiography (3D-RA). Patients were divided into the BAF group and control group (i.e., patients with the normal vertebrobasilar system). Hemodynamic and geometric variables were calculated and compared between groups using Student's *t*-test or Wilcoxon rank-sum test.

**Results:** Overall, 24 patients were included, with 12 patients each in the BAF group and the control group. The BAF group had a significantly smaller basilar artery diameter than the control group (3.1 ± 0.51 vs. 3.76 ± 0.4, *p* = 0.002). Compared to the control group, the BAF group had higher values of maxOSI (median, 0.3 vs. 0.09, *p* = 0.028), TAWSSG (median, 983.42 vs. 565.39, *p* = 0.038) in the flow confluence, higher SAR-TAWSSG in bifurcation (median, 70.22 vs. 27.65, *p* = 0.002) and higher SAR-TAWSSG in basilar artery (median, 48.75 vs. 16.17, *p* < 0.001) of the vertebrobasilar artery.

**Conclusions:** This pilot study suggested that hemodynamic differences between BAF and normal vertebrobasilar artery across multiple shear flow parameters. The disturbed flow in the BAF may increase the risk of thrombus formation, plaque instability, and subsequent ischemic cerebrovascular events. These should be confirmed by future studies.

## Introduction

Artery fenestration is a rare congenital vascular dysplasia caused by incomplete fusion of primitive embryologic vessels, which are divided into two separate channels and converge at the distal end (1). Fenestration is a developmental vascular anomaly that can occur in multiple cerebral arteries, especially in the anterior communicating artery and basilar artery (2). Basilar artery fenestration (BAF) is the second most common form of fenestration with a prevalence of 0.28–5.26% as reported in post-mortem studies (3), 0.3–0.6% in conventional angiography (4), and 1–2.07% in magnetic resonance angiography (MRA) (5–7). The size of the fenestration largely ranges from 1 mm to 5 mm (8), with a slit-like separation or long segment duplication (9). Given the innate small size and relatively low prevalence rate in imaging studies, BAF may be an easily overlooked cerebrovascular anomaly in clinical practice.

Although BAF is rare, its association with aneurysm formation and ischemic stroke has been reported (2, 10–12). Some cases were related to cerebral infarction and diagnosed as an embolic stroke of undetermined source as these patients usually had no vascular risk factors or other diseases. Therefore, this suggests that BAF is a possible cause of cryptogenic stroke (1, 2, 13). The exact mechanism of fenestration-related infarction remains unknown. Fenestration causes local hemodynamic changes, which may heighten the risk of ischemic stroke or infarction.

Computational fluid dynamics (CFD) is used to simulate flow patterns and analyze the relationship between diseases and hemodynamics. Previous studies have explored the pathophysiological relationship between changes in blood flow and the development of atherosclerosis (14, 15). Some researchers speculate that altered blood flow in the presence of artery fenestration promotes ischemic stroke, but the exact mechanism is not well-defined (16).

In this study, we first simulated flow dynamics of the BAF by using CFD and compared hemodynamic parameters with normal vertebral basilar artery. We hypothesized that there would be hemodynamic differences between the two groups. With this study, we hope to provide researchers and clinicians with updated references for future studies.

## Materials and Methods

### Patient Selection

The medical ethics committee of the hospital approved this study, and all patients provided written informed consent. From January 2020 to March 2021, 24 patients were included in this study, inclusive of 12 patients with BAF (BAF group) and 12 patients with a normal vertebrobasilar system (control group). The inclusion criteria were as follows: (1) patients diagnosed with BAF by digital subtraction angiography; (2) underwent 3D-rotational angiography (3D-RA) and division of the lumen was observed by virtual artery endoscopy; (3) the quality of images was adequate for CFD analysis; (4) fenestrations were located in the proximal segment of the basilar artery. The exclusion criteria were as follows: (1) BAF with pre-existing stenosis or aneurysm; (2) the quality of images was poor for CFD analysis. Patients who underwent MRA and had normal vertebrobasilar system were enrolled and included in the control group.

### Patient Groups and Imaging

**Figure 1:**
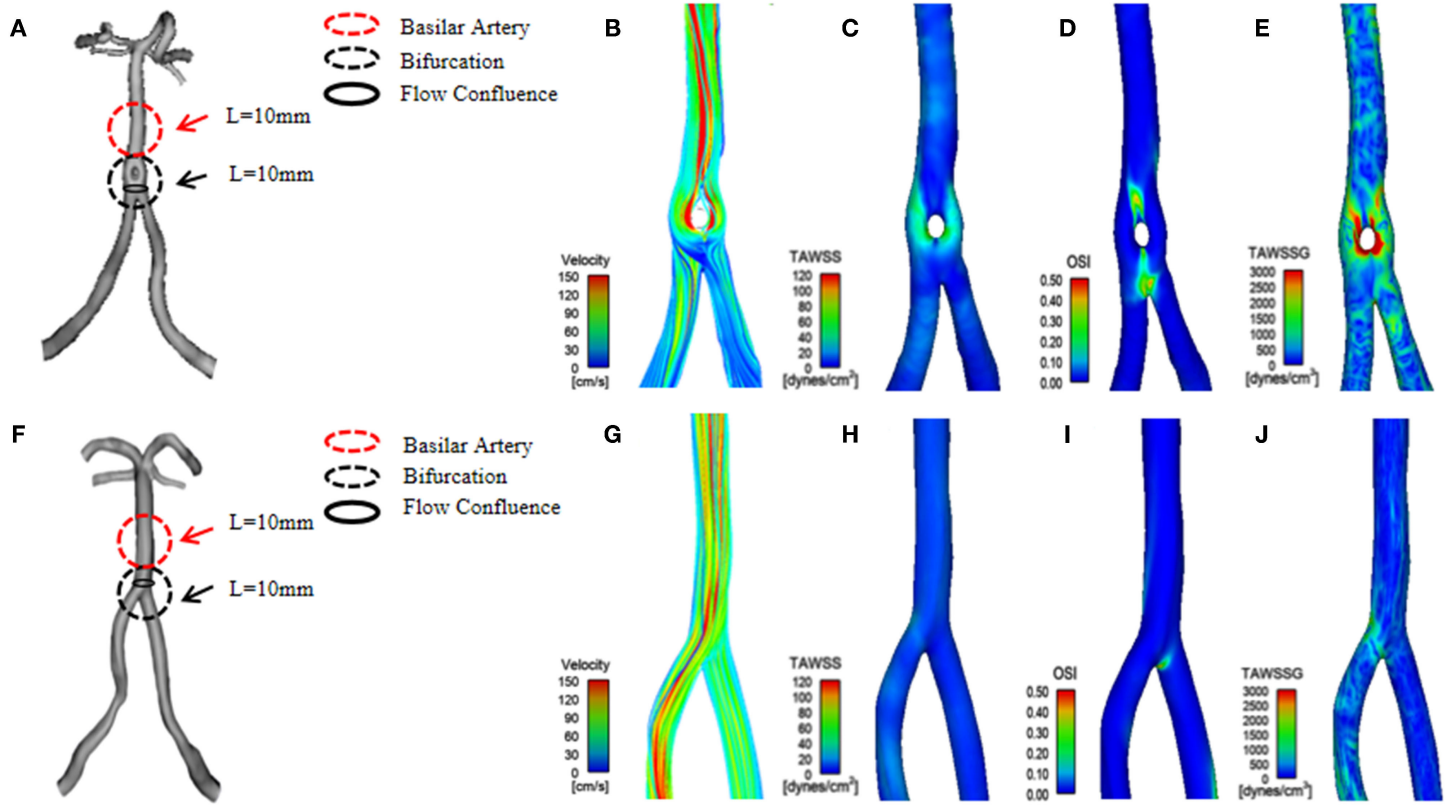
**(A)** A three-dimensional (3D) geometrical model of basilar artery fenestration (BAF) reconstructed from a patient's 3D-rotational angiography (3D-RA). **(B–E)** The hemodynamic distribution of BAF, including the following parameters: velocity, time-averaged wall shear stress (TAWSS), oscillatory shear index (OSI), and time-averaged wall shear stress gradient (TAWSSG). **(F)** A 3D geometrical model of a normal vertebrobasilar artery reconstructed from a patient's MRA. **(G–J)** The hemodynamic distribution (velocity, TAWSS OSI, and TAWSSG) of a normal vertebrobasilar artery.

To analyze the geometry and hemodynamic characteristics of a BAF, patients were divided into two groups, namely, the BAF group and the control group. Baseline data were collected, such as age, sex, smoking history, posterior circulation infarction (PCI), blood pressure level (systolic and diastolic), low-density lipoprotein (LDL), high-density lipoprotein (HDL), fasting glucose, total cholesterol, and triglycerides. This study focused on the intracranial vertebrobasilar bifurcation and basilar artery (Figure 1). In brief, 5-s rotational angiography was performed using the Innova IGS 630 biplane angiography system (General Electric, America). The protocol refers to our previous published paper (17), wherein we performed a total of 24 ml contrast agents at a flow rate of 3 ml/s, 3 s delay time, and 0.2 mm slice thickness. All patients performed virtual arterial endoscopy and confirmed the presence of fenestration. Geometric variables of interest were calculated, including the bifurcation angle, flow confluence diameter (left and right vertebral arteries merging into a basilar artery), and basilar artery diameter, tortuosity, and length. Two experienced neuroradiologists performed the reconstruction and imaging analysis.

### BAF Modeling

Three-dimensional (3D) geometry and morphometry of vertebrobasilar arteries were extracted from 3D-RA images based on thresholding using the MIMICS software (Materialize, NV, Belgium). The geometries were repaired, cut, smoothed using Geomagic Studio 2012 (3D Systems, North Carolina, USA). Then 3D models were imported into ICEM CFD (ANSYS Inc., Canonsburg, PA, United States) to generate unstructured computational meshes for simulation. All models grid ranged from 1.39 to 1.46 million elements. The wall of the vessel was assumed rigid with no-slip conditions, blood was assumed to be a laminar, incompressible Newtonian fluid. The material blood parameters were set as follows: density, *P* = 1,066 kg/m^3^; dynamic viscosity, u = 0.00345 Pa × s. ANSYS CFX 19.2 (ANSYS Inc., Canonsburg, PA, Unites States) was utilized to solve Navier-Stokes equations. The pulsatile flow was imposed at the inlet using the Womersley profile as the boundary condition. The flow waveform was derived from a patient-specific transcranial doppler (TCD) ultrasound of the vertebral artery. Outlet boundaries were set using Murray's law (18). A numerical simulation was carried out for three cardiac cycles, with a time step of 0.01 s; the results of the last cycle were used for the analysis.

### Hemodynamic Analysis

Hemodynamic parameters, time-averaged wall shear stress (TAWSS), oscillatory shear index (OSI), and TAWSS gradient (TAWSSG) were computed within the flow confluence of the vertebral artery. We further calculated surface area ratio-TAWSS (SAR-TAWSS), surface area ratio-OSI (SAR-OSI), and surface area ratio-TAWSSG (SAR-TAWSSG) in the bifurcation, basilar artery of the vertebrobasilar artery (Figure 1). Nomenclatures analyzed were as follows:

1. SAR-TAWSS in the bifurcation:

($=\frac{Surface\;area\;TAWSS\;\leq \;4dynes/cm^{2}}{bifurcation\;surface\;area}$ × 100%).

2. SAR-TAWSS in the basilar artery:

($=\frac{Surface\;area\;TAWSS\;\leq \;4dynes/cm^{2}}{basilar\;artery\;surface\;area}$ × 100%).

3. SAR-OSI in the bifurcation:

($=\frac{Surface\;area\;OSI\;\geq 0.15}{bifurcation\;surface\;area}$ × 100%).

4. SAR-OSI in the basilar artery:

($=\frac{Surface\;area\;OSI\;\geq 0.15}{basilar\;artery\;surface\;area}$ × 100%).

5. SAR-TAWSSG in the bifurcation:

($=\frac{Surface\;area\;TAWSSG\;\geq 500dynes/cm^{3}}{bifurcation\;surface\;area}$ × 100%).

6. SAR-TAWSSG in the basilar artery:

($=\frac{Surface\;area\;TAWSSG\;\geq 500dynes/cm^{3}}{basilar\;artery\;surface\;area}$ × 100%).

### Statistical Analysis

For baseline characteristics, quantitative variables are described as mean ± *SD* and qualitative variables are described as numbers and percentages. Given the non-normal distribution, the hemodynamic characteristics are presented as medians and interquartile ranges. Hemodynamic parameters of flow patterns and geometric variables were compared between BAF and control groups. Comparison of categorical variables was performed using the χ^2^ test (or Fisher's exact test as appropriate). Student's *t*-test or Wilcoxon rank-sum tests were used to comparing quantitative variables. A two-tailed *p* < 0.05 was considered significant.

## Results

### Patient and Fenestration Characteristics

The mean ± *SD* of the patient age in the BAF and control groups were 55.58 ± 12.24 and 55.25 ± 9.64, respectively (*p* = 0.942). No significant differences were found in the baseline characteristics between the two groups, including sex distribution (*p* = 1), smoking history (*p* = 1), posterior circulation infarction (*p* = 0.478), blood pressure (systolic, *p* = 0.839, diastolic, *p* = 0.708), LDL (*p* = 0.27), HDL (*p* = 0.302), fasting glucose (*p* = 0.22), cholesterol (*p* = 0.379), and triglyceride (*p* = 0.818). These data are summarized in Table 1.

**Table 1:** Patient baseline characteristics between the BAF and control group.

**Variable**	**BAF (*n =* 12)**	**Control group (*n =* 12)**	***P*-value**
Age (mean, SD)	55.581 ± 2.24	55.259 ± .64	0.942
Male, N (%)	9 (75)	10 (83)	1.000
Smoking history, N (%)	6 (50)	5 (41.67)	1.000
PCI,N(%)	2 (16.6)	0	0.478
Blood pressure, mm Hg			
SBP	128.66 ± 15.25	130 ± 16.54	0.839
DBP	75.41 ± 9.27	76.50 ± 3.50	0.708
LDL, mmol/l	2.23 ± 0.61	2.53 ± 0.64	0.270
HDL, mmol/l	1.18 ± 0.27	1.06 ± 0.26	0.302
Fasting glucose, mmol/l	5.40 ± 1.08	4.83 ± 1.11	0.220
Total cholesterol, mmol/l	3.92 ± 0.70	3.71 ± 0.41	0.379
Triglycerides, mmol/l	1.11 ± 0.38	1.15 ± 0.56	0.818

*Continuous variables are expressed as mean ± SD*

*Categorical variables are expressed as N (%)*

*PCI, posterior circulation infarction; SBP, systolic blood pressure; DBP, diastolic blood pressure; LDL, low-density lipoprotein; HDL, high-density lipoprotein*.

### BAF Group vs. Control Group

**Figure 2:**
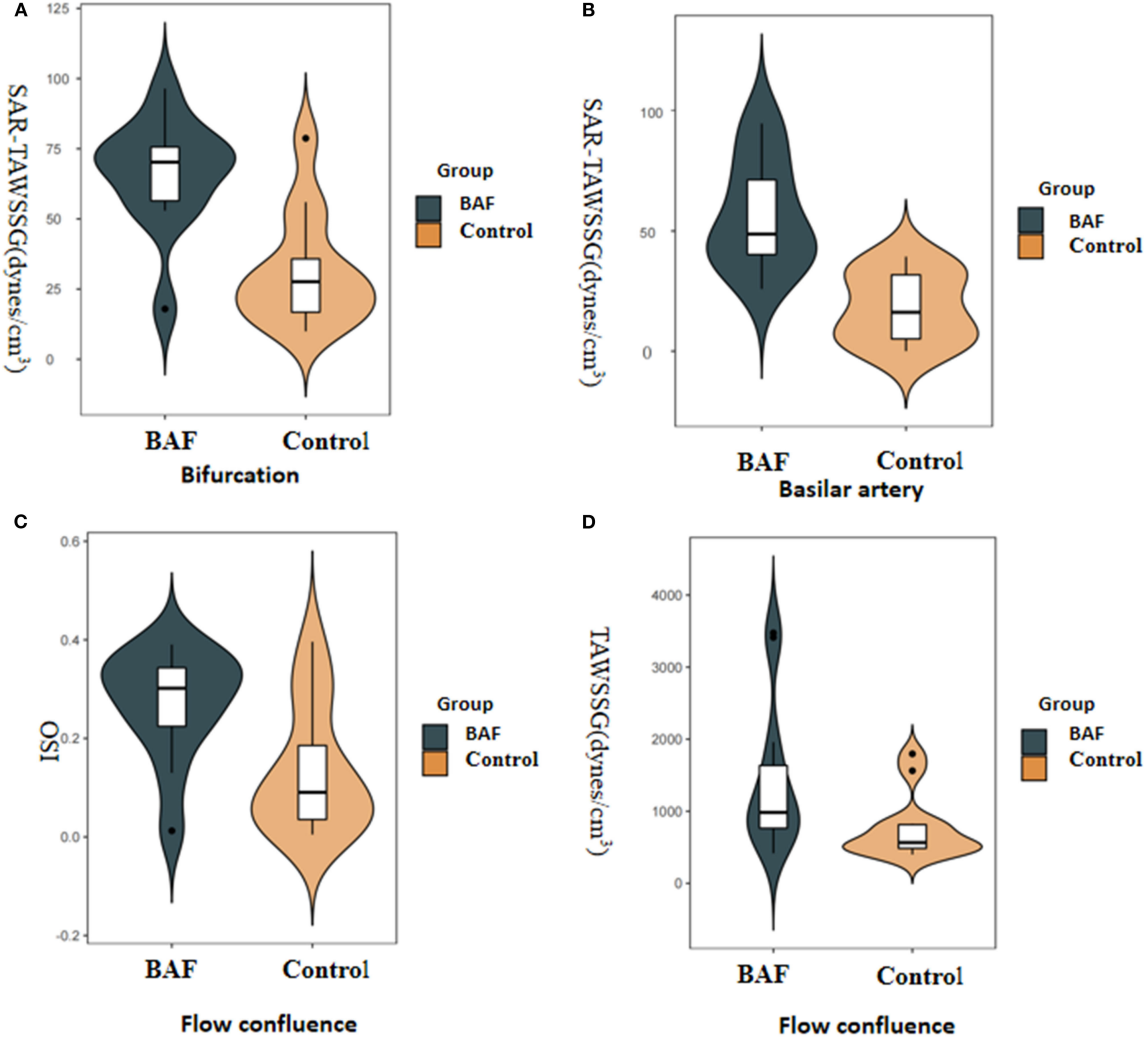
Boxplots displaying the following hemodynamic parameters: surface area ratio-TAWSSG (SAR-TAWSSG), OSI, and TAWSSG for the patients with BAF and those with a normal vertebrobasilar artery (control group). The *P*-values were obtained from the Wilcoxon rank-sum test. The BAF group had a significantly higher SAR-TAWSSG value in the bifurcation **(A)** and basilar artery zones **(B)** than the control group. The BAF group had significantly higher maximum OSI **(C)** and TAWSSG values **(D)** than the control group in flow confluence.

The values of shape, geometric features, and hemodynamics of the BAF and normal vertebrobasilar artery are provided in Table 2. Patients in the BAF group had a smaller basilar artery diameter than that those in the control group (3.1 ± 0.51 vs. 3.76 ± 0.4, *p* = 0.002). No significant differences were found between the two groups regarding any other geometric or shape parameters, including VA diameter, bifurcation angle, basilar artery curvature, and basilar artery length. When comparing hemodynamic parameters between the two groups, maximum OSI (median, 0.3 vs. 0.09, *p* = 0.028), TAWSSG (median, 983.42 vs. 565.39, *p* = 0.038) was higher at the flow confluence of BAF than the normal vertebrobasilar artery. SAR-TAWSSG was higher in bifurcation (median, 70.22 vs. 27.65, *p* = 0.002) and basilar artery (median, 48.75 vs. 16.17, *p* < 0.001) of BAF compared with normal vertebrobasilar artery. Data summarizing these are shown in Figure 2.

**Table 2:** Results of hemodynamic and morphological parameters.

**Variable**	**BAF group**	**Control group**	**P–values**
**Hemodynamics**			
Bifurcation			
SAR–TAWSS	4.85 (2.66–8.63)	3.56 (1.53–11.43)	0.670
SAR–OSI	3.03 (1.35–4.80)	0.57 (0.31–0.49)	0.184
SAR–TAWSSG	70.22 (54.16–76.56)	27.65 (15.79–44.53)	0.002*
Basilar artery			
SAR–TAWSS	0.01 (0–2.44)	0.32 (0–2.89)	0.627
SAR–OSI	0.20 (0–3.31)	0 (0–0.05)	0.123
SAR–TAWSSG	48.75 (39.62–75.57)	16.17 (4.84–32.49)	<0.001*
Flow confluence			
TAWSS (dynes/cm^2^)	26.75(18.07–48.16)	20.64 (13.85–24.46)	0.106
maxOSI	0.30 (0.22–0.34)	0.09 (0.03–0.24)	0.028*
TAWSSG (dynes/cm^3^)	983.42 (761.74–1851.10)	565.39 (474.87–823.10)	0.038*
**Geometry**			
VA diameter difference (mm)	0.43 (0.29–1.00)	0.47 (0.15–0.94)	0.664
Basilar artery curvature	0.05 (0.04–0.07)	0.03 (0.02–0.06)	0.244
Flow confluence diameter (mm)	4.81 ± 0.86	4.28 ± 0.48	0.073
Bifurcation angel (degree)	65.57 ± 13.14	64.88 ± 9.39	0.882
Basilar artery diameter (mm)	3.10 ± 0.51	3.76 ± 0.40	0.002*
Basilar artery length (mm)	26.48 ± 2.92	25.11 ± 3.35	0.297

*Medians and interquartile ranges (IQR) due to a non-normal distribution.*

*Continuous variables are expressed as mean ± SD.*

*TAWSS, time-averaged wall shear stress; OSI, oscillatory shear index; TAWSSG, time-averaged wall shear stress gradient; SAR-TAWSS, surface area ratio -time-averaged wall shear stress; SAR-OSI, surface area ratio-oscillatory shear index; SAR-TAWSSG, surface area ratio-time-averaged wall shear stress gradient.*

*p < 0.05.

## Discussion

This study provides morphometry and flow patterns associated with BAF in vertebrobasilar arteries through CFD. We observed that patients with BAF had higher OSI, TAWSSG, and SAR-TAWSSG values than those of control subjects with normal vertebrobasilar arteries. Furthermore, at the flow confluence, higher OSI and TAWSSG were observed in the proximal segment of patients with BAF. These results verified that fenestration could disturb the hemodynamic patterns in relevant anomalous arteries. Thus, more attention should be paid to this rare congenital vascular disease in clinical settings.

Various CFD parameters are associated with atherosclerosis (14, 19). WSS is used to describe the tangential friction of blood flow on the vascular wall and is known to affect the biological function of endothelial cells (20, 21). Low WSS stimulates atherosclerosis in its early stage, while high WSS can cause endothelial trauma, modulate plaque composition, and reduce cap stability thereby promoting acute thrombus formation upon plaque rupture (22, 23). High WSS is usually accompanied by spatial WSSG in complex vascular geometries. Endothelial cells are more sensitive to WSSG and have different response gradient signals (24). Therefore, in addition to high WSS, WSSG may contribute to alterations in the vulnerable plaque pathobiology. A recent study observed that patients with vertebral artery stenosis had significantly high SAR-TAWSSG and a high incidence of transient ischemic attacks (TIAs) or posterior circulation infarction. Further, high SAR-TAWSSG may induce TIA or posterior circulation infarction (25). In our study, we found that higher SAR-TAWSSG both at the bifurcation and basilar artery zone of patients with BAF compared to that of those patients with normal vertebrobasilar arteries. A previous study reported that BAF is a risk factor for intracranial atherosclerotic plaque, and plaques are mostly located in the proximal and bifurcation segments of BAF (26). High SAR-TAWSSG values were found in the bifurcation of patients with BAF in our study, which may be the cause of unstable plaque formation. The basilar artery is also prone to plaque formation with many perforating arteries. As hemodynamics change, BAF-induced disturbance of flow may further precipitate diffuse diseases in this vessel. Thus, both high SAR-TAWSSG and diffuse vascular diseases may increase the instability of basilar artery plaque, resulting in TIA or infarctions. However, this requires further investigation.

Our study also demonstrates that lower TAWSS is usually located at the outer wall and the proximal as well as bifurcation segments of a BAF (Figure 1). This hemodynamic phenomenon is similar to that in the branches of coronary arteries. According to hydrodynamic theory, WSS in the inner/medial wall is higher than at other components of the vessels. In contrast, the stress in the outer/lateral wall is lower, and its distribution is more irregular (27). We calculated the hemodynamic parameters on the surface of the flow confluence segment of vertebrobasilar arteries. The flow confluence of the BAF converges at the bilateral vertebral artery and then separates into two channels. In a short distance, flow velocity changes rapidly and tend to be characterized by the disturbed flow. Thus, it may create large spatial variations in WSS, which increases the risk of thrombosis formation and subsequent ischemic stroke. BAF-related infarction may be associated with local thrombosis has been reported (13). Further, an artery obstructed by a thrombus or plaque debris lodged is both scenarios that may lead to ischemic stroke. In our study, higher OSI and TAWSSG values at the flow confluence of the BAF were noted, especially in the middle sidewall (Figure 1). Plaque development is associated with low and oscillating WSS, and OSI can quantify the changes in WSS direction over the cardiac cycle. OSI values are associated with changes in endothelial gene expression, lipid accumulation, and inflammatory cell activation. Therefore, a high OSI value is considered an indicator of vascular endothelial dysfunction (28). We speculate that higher OSI and TAWSSG of the BAF can result in multidirectional blood flow disorders, which could easily cause vessel wall dysfunction, promote platelet aggregation, and eventually result in thrombus formation and plaque instability (27). Therefore, we speculate that the flow confluence segments of the BAF are high-risk locations for thrombus formation, plaque development, and rupture. Even though our study excluded BAF with stenosis or aneurysm, a numerically higher rate of posterior circulation infarction was observed in the BAF group (16.6 vs. 0%). Future studies involving more BAF patients with symptoms or serial observation of hemodynamic characteristics and the correlation with cerebrovascular events are warranted.

### Limitations

This study has several limitations. First, with the low prevalence of BAF, only 12 patients were included over the study period. Although there is a trend in the results, a small sample may have weakened our conclusion. Second, CFD is a commonly employed method used to obtain hemodynamic parameters but does inevitably involve some assumptions. Modeling assumptions may result in deviation from the actual flow as it would occur *in vivo*. Finally, a follow-up study with a larger sample size is required to more clearly establish a correlation between BAF, vessel wall changes, and the occurrence of cerebrovascular events.

### Conclusions

This pilot study suggests that there may be hemodynamic differences between BAF and normal vertebrobasilar systems. BAF results in high OSI, TAWSSG, and SAR-TAWSSG. Turbulent flow in the BAF might induce thrombus and unstable plaque formation, which increases the risk of ischemic stroke. Combined with high-resolution magnetic resonance imaging for vascular wall imaging, the hemodynamic characteristics of BAF may be more comprehensively evaluated in future studies.

## Data Availability Statement

The original contributions presented in the study are included in the article/supplementary material, further inquiries can be directed to the corresponding authors.

## Author Contributions

LJ and DC contributed to the conception and design of the study. JD drafted the initial manuscript. YM and XT performed the simulation. XB and AD wrote sections of the manuscript. BY, TW, and AP critically reviewed and revised the manuscript. XY, ML, and RY organized the database. All the authors reviewed and approved the final version of the manuscript.

## Funding

This work was supported by the Xuanwu Hospital Science Program for Fostering Young Scholars (QNPY2020003) and Beijing Natural Science Foundation (Z190014). The funders have no role in study design, data analysis, and writing for the manuscript.

## Conflict of Interest

The authors declare that the research was conducted in the absence of any commercial or financial relationships that could be construed as a potential conflict of interest.

## Publisher's Note

All claims expressed in this article are solely those of the authors and do not necessarily represent those of their affiliated organizations, or those of the publisher, the editors and the reviewers. Any product that may be evaluated in this article, or claim that may be made by its manufacturer, is not guaranteed or endorsed by the publisher.
